# Quantitative MR Analysis of Changes in the Radius Bone Marrow in Osteoporosis

**DOI:** 10.1155/2023/7861495

**Published:** 2023-12-27

**Authors:** Tamar K. De-Levie, Yael S. Schiffenbauer, Ido Druckmann, Vanessa Rouach, Naftali Stern, Itzhak Binderman, Uri Nevo

**Affiliations:** ^1^Department of Biomedical Engineering, Tel Aviv University, Tel Aviv, Israel; ^2^Faculty of Medicine, Tel Aviv University, Tel Aviv, Israel; ^3^Skeletal Imaging Division, Tel Aviv Sourasky Medical Center, Tel Aviv, Israel; ^4^Institute of Endocrinology, Metabolism and Hypertension, Tel Aviv Sourasky Medical Center, Tel Aviv, Israel; ^5^The Sagol Center for Epigenetics, Tel Aviv Sourasky Medical Center, Tel Aviv, Israel; ^6^The Sagol School of Neuroscience, Tel Aviv University, Tel Aviv, Israel; ^7^Department of Oral Biology, School of Dental Medicine, Tel Aviv University, Tel Aviv, Israel

## Abstract

**Purpose:**

This pilot study aimed to explore the feasibility of scanning the human distal radius bone marrow in vivo to detect osteoporosis-related changes using magnetic resonance and evaluate whether the radius may serve as an accessible probing site for osteoporosis. This may lead in the future to the use of affordable means such as low-field MRI scanners for the monitoring of disease progression.

**Methods:**

A clinical trial was performed using a 3T MR scanner, including 26 women assigned into three study groups: healthy-premenopausal (*n* = 7; mean age 48.6 ± 3.5 years), healthy-postmenopausal (*n* = 10; mean age 54.5 ± 5.6 years), and osteoporotic-postmenopausal (*n* = 9; mean age 61.3 ± 5.6 years). Marrow fat composition was evaluated using T2 maps, a two-compartment model of T1, and a Dixon pulse sequence.

**Results:**

The osteoporotic group exhibited higher fat content than the other two groups and lower T2 values than the healthy-premenopausal group.

**Conclusions:**

Osteoporosis-related changes in the composition of the distal radius bone marrow may be detected in vivo using MRI protocols. The scanning protocols chosen here can later be repeated using low-field MRI scanners, thus offering the potential for early detection and treatment monitoring, using an accessible, affordable means that may be applied in small clinics. This trial is registered with MOH_2018-05-23_002247, NCT03742362.

## 1. Introduction

Osteoporosis is a metabolic disease characterized by deterioration of the microarchitecture of the bone favoring osteoclastic over osteoblastic activity, leading to a decline in bone mineral density (BMD), thus making it more susceptible to fractures [1].

The clinical manifestations of osteoporosis are fractures generated by low-energy trauma, one of the hallmark fracture sites being the distal radius [2, 3]. The World Health Organization (WHO) operationally defines osteoporosis by the value of BMD as measured via dual-energy X-ray absorptiometry (DEXA). Thus, a T-score of −2.5 or lower defines the presence of osteoporosis, meaning 2.5 standard deviations (SD) below the mean for young healthy adults of the same sex and race [4].

Consistently, alterations in the composition of the bone marrow are also evident in osteoporosis, owing to a shift in the differentiation of mesenchymal stem cells, favoring adipocytes over osteoblasts [5]. Numerous studies have shown that BMD is inversely correlated to the bone marrow fat fraction. Furthermore, changes in the bone marrow adiposity are considered to precede the changes detected in BMD [6–9].

Previous studies have shown that MRI can be used to detect changes in the bone marrow fat fraction of the axial skeleton, primarily the lumbar spine and the femoral neck using the Dixon protocol [10, 11]. Other studies have shown that MRI can discriminate between healthy, osteopenic, and osteoporotic-postmenopausal women based on the internal magnetic field gradient in calcaneus cancellous bone [12]. MRI also showed a correlation of the trabecular bone structure and osteoporosis status in the distal radius [13].

Single-voxel MR spectroscopy (MRS) has been utilized in several studies to quantify bone marrow fat in the axial skeleton [11, 14, 15], as a biomarker for osteoporosis and an indicator of treatment efficacy [9]. Nevertheless, its application in the peripheral skeleton, particularly in the upper extremities, remains relatively unexplored.

Pietro et al. have highlighted the potential of MRS in the femoral neck bone marrow as a promising screening tool for osteoporosis [16]. Other studies have explored alterations in bone marrow composition of the lower extremities in conditions such as Crohn's disease and anorexia nervosa [17, 18]. These analyses indicate that while the bone marrow in the extremities is inherently lipid-rich, MR can discern changes in fat composition, offering potential markers for various pathologies associated with a higher risk of osteoporosis.

Despite its obvious advantages, including the lack of ionizing radiation and good tissue-differentiation capacity, MRI remains a relatively nonaccessible imaging modality for the assessment of bone marrow changes in osteoporosis, mainly due to its high price tag.

A possible mitigation strategy may be the use of low-cost low-field MRI systems. Indeed, Sarda et al. and Hillel et al. [19, 20] used animal models and suggested that stray field NMR can be used to detect changes in bone marrow composition, potentially enabling screening and treatment follow-up [19, 20]. Importantly, translation of such works to humans requires a target bone that can easily be probed using such scanners.

The following study explores the feasibility of scanning the human distal radius in vivo using magnetic resonance to detect osteoporosis-related changes in bone marrow composition. The incentive for this exploration is to evaluate whether the radius may serve as an accessible site, allowing scanning and monitoring using a simple and affordable means such as low-field MRI scanners. Preliminary results of this study were already presented in the 31^st^ annual meeting of the ISMRM [21].

## 2. Methods

### 2.1. Study Protocol and Design

A total of 26 women were recruited by the Institute of Endocrinology at the Tel Aviv Sourasky Medical Center (Tel Aviv, Israel) for the present study. Prior to the study, all subjects underwent a dual-energy X-ray absorptiometry (DEXA) scan to measure bone mineral density (BMD) and were assigned into three study groups according to their menopausal status and T-scores, as follows: healthy-premenopausal (*n* = 7, mean age 48.6 ± 3.5 years), healthy-postmenopausal (*n* = 10, mean age 54.5 ± 5.6 years), and osteoporotic-postmenopausal (*n* = 9, mean age 61.3 ± 5.6 years) patients (additional information regarding the group division and clinical data of the participants are found in the supplementary materials (available here)). Blood serum concentrations of bone turnover markers and hormonal blood profiles were tested as well, including FSH, LH, estradiol 17 beta, procollagen type 1 N-terminal propeptide (P1NP), and C-terminal crosslinking telopeptide of collagen type I (CTX). All the subjects were otherwise healthy as determined by medical history and physical examination. The exclusion criteria included pregnancy, metabolic diseases, diabetes, the use of hormone-based contraceptives or replacement, long-term steroids or osteoporosis treatment, BMI bellow 20 or above 27, and contraindications to MRI such as metal implants and tattoos in the area of interest.

The study was approved by the Ethics Committee of the Tel Aviv Sourasky Medical Center and Tel Aviv University, and informed consent was obtained from all the subjects prior to the examination.

### 2.2. MRI Scan Protocol

All MRI scans were performed at the Alfredo Federico Strauss Imaging Center in Tel Aviv University using a Siemens Magnetom Prisma 3T whole-body scanner equipped with a 4-channel flex coil. In all the imaging scans, slice thickness was 4.5 mm and in-plane resolution was 0.85 mm.

#### 2.2.1. Bone Localization

For each participant, a pilot scan in 3 orthogonal planes was performed in order to localize the anatomical region of interest (ROI) to include the metaphysis area.

A T2-weighted single-shot turbo spin echo (TE 103 msec; TR 1200 msec) was used to rule out pathologies and as the basis for localization of single-voxel spectroscopy.

#### 2.2.2. Spectroscopy

Single-voxel spectroscopy (SVS) was performed using a PRESS type pulse sequence with the following scan parameters: TE 14 msec, TR 1500 msec, and BW 2000 Hz, in a voxel of 1 × 1 × 1 cm^3^. The voxel was located at the region between the epiphysis and the metaphysis where the bone narrows. However, due to the limited spatial resolution dictated using our scanner, the voxel included tissues other than the bone marrow and the compact bone, thus potentially altering the fat-water composition inconsistently. The spectroscopy results were therefore excluded.

#### 2.2.3. T1 Measurement

T1 maps were obtained using a turbo spin echo (TSE) sequence with 7 different TRs between 416 and 5500 msec and TE 8.5 msec.

#### 2.2.4. T2 Measurement

T2 maps were obtained using a multislice multiecho (MSME) sequence with 22 different TEs between 12 and 264 msec and TR 3659 msec.

#### 2.2.5. Fat Separation (Dixon)

The bone marrow fat fraction (BMFF) of the distal radius was measured using a T1 weighted two-point VIBE Dixon sequence, with a TR of 5.23 msec, a TE of 2.46 and 3.69 msec, and a flip angle of 9°.

Of the 26 woman who participated in the clinical trial, one postmenopausal woman with osteoporosis had missing T1 and 3 women from the healthy-postmenopausal group had missing Dixon data, all due to acquisition artifacts.

### 2.3. DEXA

As previously mentioned, the diagnosis of osteoporosis requires a T-score of −2.5 or lower in at least one of the anatomical sites scanned, regardless of the T-scores of the other anatomical sites. DEXA scans included three anatomical sites of the axial skeleton (the lumbar spine, femoral neck, and total hip), recorded as three separate T-score values.

### 2.4. Data Analysis

Data blinding was conducted at the time of acquisition so that the analysis was blinded to participants.

A T2-weighted scan of each examinee was evaluated by a radiologist to rule out pathologies.

**Figure 1:**
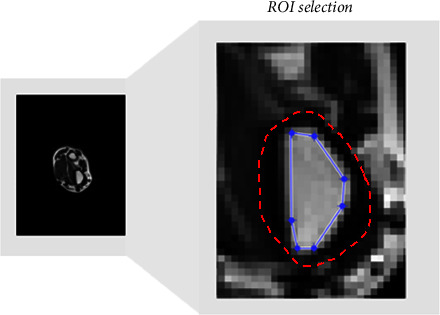
ROI was selected manually per slice to include the bone marrow of the metaphysis area of the radius. ROI of the bone marrow is marked by a dotted line. The compact bone is marked by a dashed line.

For each scanning protocol, three slices covering the metaphysis area were manually selected per examinee. The region of interest (ROI) of the bone marrow was manually defined per slice, using a graphic cursor, sparing the interface between the bone marrow and the compact bone, to avoid edge effects (Figure 1). Thus, the ROIs exclusively consisted of the bone marrow within niches of trabecular bone.

#### 2.4.1. Extraction of T1 Maps

A two-compartment model was applied referring to water and fat as two separate compartments of the bone marrow. The fat fraction (FF) and T1 relaxometry values of the fat (*T*1_*f*_) and water (*T*1_*w*_) compartments were calculated using biexponential fit according to the following equation:

(1) $$\begin{array}{c} s=s_{0}∙\left(\text{FF}∙\left(1-e^{-t/{T1}_{f}}\right)+\left(1-FF\right)∙\left(1-e^{-t/{T1}_{w}}\right)\right), \end{array}$$

where *S* is the detected magnetization intensity, *s*_0_ is the equilibrium magnetization parallel to the main static magnetic field *B*_0_, FF is the fat fraction of the specimen, *T*1_*f*_ is the longitudinal relaxation time constant of the fat compartment, and *T*1_*w*_ is the longitudinal relaxation time constant of the water compartment. In the fitting process, *T*1_*w*_ was set within the range of 1050–1250 msec and *T*1_*f*_ in the range of 350–390 msec. These values were chosen with reference to the work of Neumayer et al. that applied a two-compartment model of the human bone marrow [22]. The combination of relaxometry values providing the highest *R*^2^ value was chosen. The fit was made over the spatial ROI including all three slices per volunteer.

#### 2.4.2. T2 Measurement

The T2 relaxometry value was calculated using the mono-exponential fit (a biexponential fit with two compartments was also tested, but it showed no superiority in terms of goodness of fit). The fit was made over the spatial ROI including all three slices per volunteer, according to the following equation:

(2) $$\begin{array}{c} s=s_{0}∙\left(e^{-t/T2}\right)+\text{const}, \end{array}$$

where *S* is the measured magnetization intensity, *s*_0_ is the initial maximal value parallel to the main static magnetic field *B*_0_, and *T*_2_ is the relaxation time constant of the transverse components of magnetization.

#### 2.4.3. Fat Separation

Dixon is a chemical-shift based imaging method, in which four sets of images are acquired: an in-phase (IP) image, containing the sum of fat and water signals, an opposed-phase (OP) image, containing the difference between water and fat signals, a fat-only image, and a water-only image, both derived from the mathematical subtraction or summation of the IP and OP images, respectively. The fat-only image offers the potential for fat quantification.

From the acquired Dixon images, a fat fraction image was calculated according to the following equation:

(3) $$\begin{array}{c} \text{FF}=\frac{F}{W+F}, \end{array}$$

where FF is the fat fraction of the specimen, *F* is the fat signal, and *W* is the water signal. The average BMFF at the ROI was computed per slice, excluding outliers more than 2 standard deviations from the mean value, and an average value over all three slices was calculated per volunteer.

### 2.5. Statistical Analysis

A single-tailed *T*-test was conducted to determine the significance of the age difference between the groups.

One-way ANOVA and ordinal regression were conducted to compare the effect of independent continuous variables obtained by MR scans (T2, fat fraction calculated using T1 and fat percentage obtained by the Dixon protocol) on the trial group division.

*p* values <0.05 were considered statistically significant.

BMD of the radius bone itself was not scanned by DEXA, and in the absence of a pre-existing weighted T-score in clinical use, we sought to create an additional group division in which the three T-scores are weighed, in order to better evaluate the true correlation between the BMD and MR parameters of the bone marrow of the radius. Therefore, we generated novel group assortment considering the combination of the three T-scores, creating an ordinal variable of 4 levels. Further details regarding the group assortment are described in Supplementary Materials.

An additional ordinal regression model was conducted, with four independent continuous variables: T2, fat fraction calculated using T1, fat percentage obtained by the Dixon protocol, and age. The dependent variable was the new T-score-based group assortment. For multidimensional analysis, we applied the principal component analysis (PCA) to facilitate the interpretation of the results.

## 3. Results

### 3.1. Groups May Be Distinguished Using the Dixon Contrast

**Figure 2:**
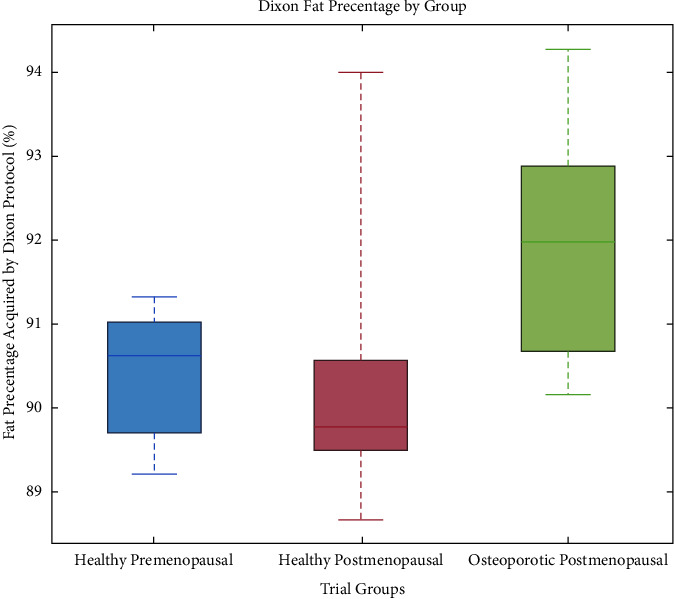
Distribution of fat percentage values of the radius BM according to the trial groups, as acquired by the Dixon protocol. Fat percentage varied as follows: healthy-premenopausal: 90.43 ± 0.82 (%), healthy-postmenopausal: 90.37 ± 1.72 (%), and osteoporotic-postmenopausal: 91.99 ± 1.38 (%).

**Figure 3:**
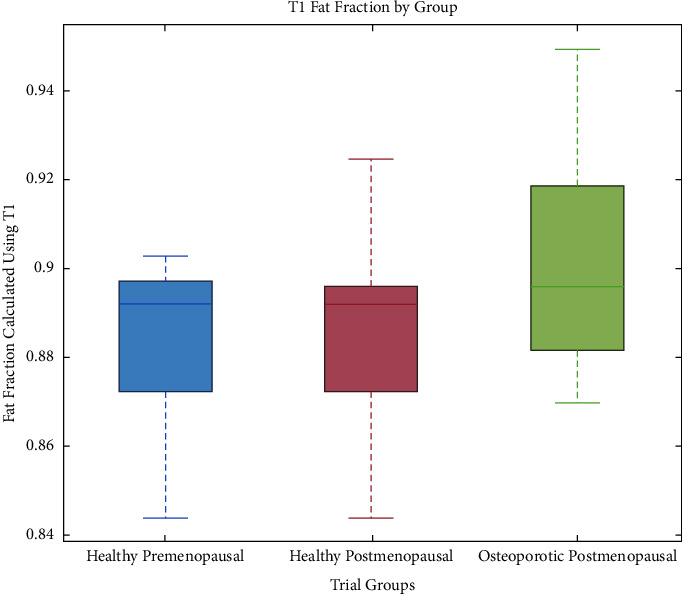
Distribution of fat fraction values of the radius BM, obtained by a two-compartment model of T1 relaxation time. Fat fraction varied as follows: healthy-premenopausal: 0.88 ± 0.02, healthy-postmenopausal: 0.89 ± 0.02, and osteoporotic-postmenopausal: 0.90 ± 0.03.

**Figure 4:**
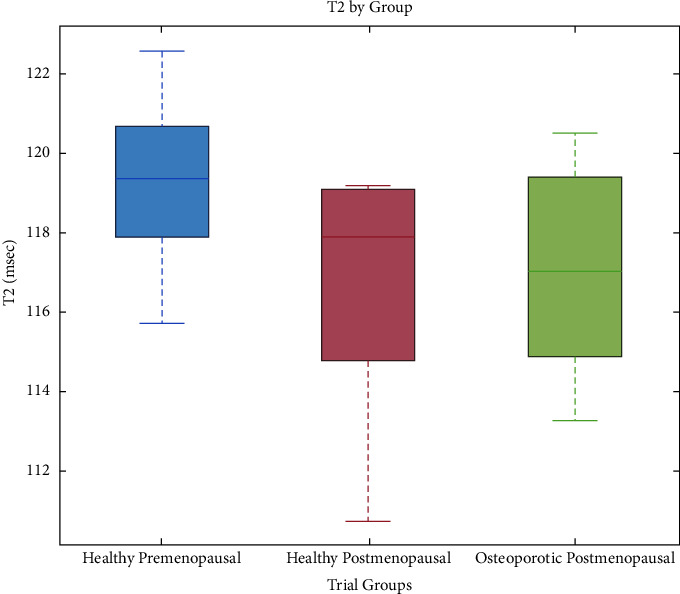
Distribution of T2 values of the radius BM by trial groups. T2 values varied as follows: healthy-premenopausal: 119.35 ± 2.27 (msec), healthy-postmenopausal: 116.71 ± 3.01 (msec), and osteoporotic-postmenopausal: 117.07 ± 2.72 (msec).

Figures 2–4 depict the distributions of the group values of the fat fraction, from each of the measurements. In all figures, the central mark indicates the median and the bottom and top box-edges indicate the 25th and 75th percentiles, respectively. Whiskers extend to the most extreme data points. No outliers were removed.

Figure 2 shows the distribution of fat percentage values according to the trial groups, as acquired by the Dixon protocol. The mean group values for the fat percentage varied as follows: healthy-premenopausal: 90.43 ± 0.82 (%), healthy-postmenopausal: 90.37 ± 1.72 (%), and osteoporotic-postmenopausal: 91.99 ± 1.38 (%).

### 3.2. Groups May Be Distinguished by T1 Values

Figure 3 shows the distribution of fat fraction values obtained by a two-compartment model of T1. The mean group values for the fat fraction varied as follows: healthy-premenopausal: 0.88 ± 0.02, healthy-postmenopausal: 0.89 ± 0.02, and osteoporotic-postmenopausal: 0.90 ± 0.03.

### 3.3. Groups May Be Distinguished by T2 Values

Figure 4 shows the distribution of T2 values by trial groups. Of the 26 woman who participated in the clinical trial, 2 women had missing T2 data due to acquisition artifacts—one healthy-postmenopausal woman and one postmenopausal woman with osteoporosis. T2 values varied as follows: healthy-premenopausal: 119.35 ± 2.27 (msec), healthy-postmenopausal: 116.71 ± 3.01 (msec), and osteoporotic-postmenopausal: 117.07 ± 2.72 (msec).

### 3.4. Combination of MR Parameters May Distinguish between Groups

**Figure 5:**
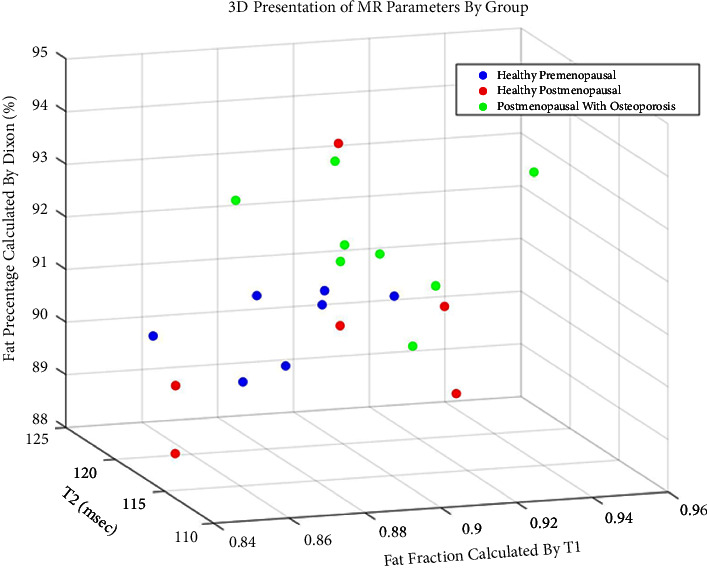
Three-dimensional presentation of the MR measurements of the radius BM distributed per group, including fat percentage calculated using the Dixon protocol, fat fraction obtained using a two-compartment model of T1 relaxation time, and T2 values.

A three-dimensional presentation of the results is shown in Figure 5. The healthy-premenopausal group is clearly distinguished from the group of postmenopausal women with osteoporosis, whereas the results of the healthy-postmenopausal group when grouped with the healthy-premenopausal (blue + red) seem to segregate from the osteoporosis group (green).

**Figure 6:**
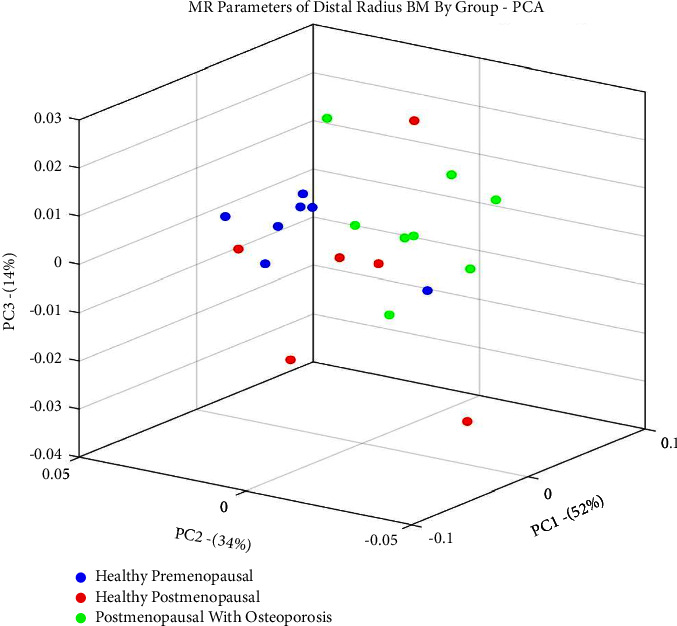
Principal component analysis (PCA) shows distinct separation between the healthy-premenopausal group and the group of postmenopausal women with osteoporosis.

Principal component analysis (PCA) results are shown in Figure 6, demonstrating the same trend of distinct separation between the healthy-premenopausal group and the group of postmenopausal women with osteoporosis.

### 3.5. Radial Bone Marrow Fat Content Correlates with Axial Bone Density

**Figure 7:**
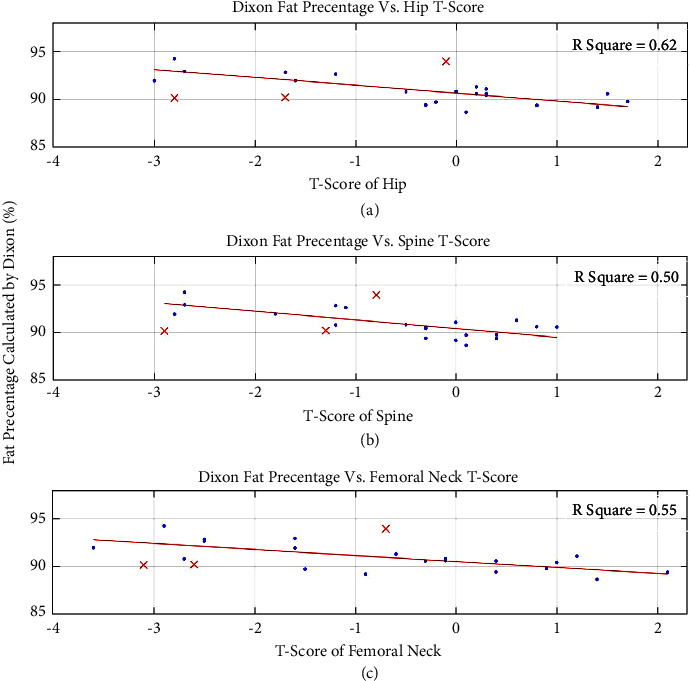
Correlation between fat percentage calculated using the Dixon protocol and T-scores of the axial skeleton measured by DEXA. The acquired data were fitted using linear least squares in MATLAB (MathWorks, MA). Outliers not included in the fit are marked (red crosses). *R*^2^ value of each fit is noted. A linear correlation is evident.

Figure 7 shows the correlation between fat percentage values acquired using the Dixon protocol and the separate axial T-scores of each examinee. A linear trend is evident.

### 3.6. Results of Statistical Analysis

Age differences between the healthy-premenopausal and both the healthy-postmenopausal and osteoporotic-postmenopausal groups were statistically significant (*p* < 0.01, *p* < 0.001, respectively).

Due to partially missing data owing to technical acquisition artifacts, only 21 of the 26 cases were included in the regression model, resulting in a relatively small sample size.

One-way ANOVA revealed that there was a statistically significant difference in the fat percentage obtained by the Dixon protocol between at least two groups (*F* (2, 20) = 3.715; *p*=0.04).

Post hoc analysis using the LSD test for multiple comparisons found that the mean value of the Dixon fat percentage was significantly different between the healthy-premenopausal group and the osteoporotic group (*p*=0.03; 95% C.I. = [−2.98, −0.12]) and between the healthy-postmenopausal group and the osteoporotic group (*p*=0.03; 95% C.I. = [−3.05, −0.19]).

There was no statistically significant difference between the healthy-premenopausal group and the healthy-postmenopausal group (*p*=0.93).

No colinearity problem was detected using the ordinal regression model. The assumption of proportional odds was met, as assessed by a full likelihood-ratio test comparing the fit of the proportional odds model to a model with varying location parameters (*χ*^2^ (8) = 8.620; *p*=0.636). None of the independent variables was found as significantly related to the trial group assortment (*p* values =0.13, 0.25, and 0.11) possibly due to the small sample size.

An attempt to obtain additional statistical analysis with a weighed T-score as the dependent variable has not yielded significant results. Once again, 21 of the 26 cases were included in the regression model, and no colinearity problem was detected. Of the 4 independent variables included in the model, age was the only one found as significantly related to the T-score group assortment (*p* value =0.043).

## 4. Discussion

This study is meant to evaluate whether changes in the composition of the bone marrow of osteoporotic women may be detected in vivo using MR scans of the radius bone. The MRI scanning protocols chosen in the study design are the ones that could later be recreated using low-field MRI scanners. These may potentially offer an accessible, affordable means of an early detection and monitoring of treatment that may even be applicable used in small clinics. The results reflect the changes detected in the bone marrow's composition in the metaphysis of the radius bone [23].

### 4.1. Physiological Correlates of the Results

Our MR scan results included T2 measurement, fat fraction calculation based on a two-compartment model applied on T1 measurement, and fat percentage obtained by the fat separation technique using the Dixon protocol.

A consistent trend is seen, in which the measured variables are distributed more homogeneously in the healthy-premenopausal group than in both the healthy-postmenopausal and the osteoporotic-postmenopausal groups. This is reflected in a relatively lower standard deviation of the results of the healthy-premenopausal group in all parameters. As expected, the osteoporotic-postmenopausal group shows higher median values for the fat percentage (Figure 2) and fat fraction (Figure 3) and lower median T2 values (Figure 4) than the healthy-premenopausal group. Those results may be easily explained by the higher fat content of the fatty bone marrow associated with osteoporosis. Nevertheless, the healthy-postmenopausal group results are less consistent, with a wider diversity and a lower average T2 value than in the other two groups. This finding, along with the previously mentioned trend, may be attributed to the pathological changes that are seen in the microstructure of aging bones. With age, two processes occur simultaneously: fat accumulation in the bone marrow along with marked thinning and increased porosity of the bone matrix resulting in a decreased surface-to-volume ratio in the medullary cavity. As hypothesized by Sarda et al., the two processes have a potentially opposite effect on MR parameters such as T2, as fat accumulation potentially lowers T2, whereas the low surface-to-volume ratio within the pores potentially increases T2 [19]. Moreover, physiological and pathological conditions, oedema, or bone marrow lesions may also affect the bone marrow's composition. These may be linked to lifestyle features that were not incorporated in our exclusion criteria, such as diet or sedentary lifestyle. Those changes in the bone marrow may sometimes serve as an isolated finding or precede a later clinical diagnosis, as it may be present in very early stages of several pathologies such as rheumatic diseases [24]. The effect of such pathological conditions on the composition of the bone marrow also implies further potential of the aforementioned protocols in the early detection and characterization of bone marrow lesions and in guiding a biopsy or planning a surgery. Of course, it should be noted that our relatively small sample size may have also affected the apparent trends, and therefore, a further investigation of a larger scale is recommended.

### 4.2. Limitations

A main limitation of the current study is its relatively small sample size, limited by resources, exclusion criteria, and dropout of volunteers. Moreover, as age seems to have a considerable effect on both BMD and bone marrow composition, a more distinct difference of age distribution between the trial groups might have been preferable.

Regarding the study design, all women went through DEXA scans of 3 axial anatomical sites (the lumbar spine, total hip, and femoral neck). We recommend that future studies should include reference measurement of the BMD of the radius bone as well.

Previous studies have shown BMD is inversely related to the bone marrow fat fraction [6–9]. Yet, in the absence of a pre-existing weighted T-score, which is based on measurements from multiple sites, in clinical use, the correlation between the BMD of the distal radius and the axial T-scores remains ambiguous. Moreover, some nonosteoporotic-postmenopausal women were found to exhibit bone loss preferentially at the distal part of the radius, which may result in fragility fractures in that area [25].

Prior research has demonstrated that quantitative assessment of the trabecular bone density of the distal radius using MRI exhibited reasonable reproducibility in vivo [26], and in studies of larger cohorts, more scans can be useful to assess reproducibility of the results, as well to assess possible bias of the results, due to the manual selection of ROIs for analysis. Furthermore, considering previous studies highlighting the different behavior of bone marrow fat accumulation in varied peripheral anatomical sites [27], we suggest performing synchronous scans of both the distal radius and the lumbar spine of the same examinee in order to evaluate the concordance between MR parameters of the bone marrow of the different anatomical sites.

## 5. Conclusions

In this study, we show that a notable separation is apparent between the healthy-premenopausal group and the osteoporotic-postmenopausal group in both the 3D presentation and PCA. This, along with the evident correlation between the Dixon obtained fat percentage and axial skeleton T-scores, implies that the bone marrow of the distal radius may potentially serve as a probing site for early detection and monitoring of treatment for osteoporosis using MR parameters. This further implies that the simple MRI scanning protocols chosen in this study can later be repeated using low-field MRI scanners.

The diversity of the results of the healthy-postmenopausal group may be attributed to the aforementioned processes that affect the aging bone.

## Data Availability

A file including all clinical data of the participants is attached in Supplementary Materials.

## Supplementary Materials

Supplementary Materials (1) A file including all clinical data of the participants is attached. (2) Scatterplots of the data depicted in Figures 2–4 are available in a separate file, to illustrate the variations within the groups. (3) Additional group assortment by T-scores: as mentioned, in the absence of a pre-existing weighted T-score in clinical use, we sought to create an additional group division in which the three T-scores are weighed. Therefore, we generated novel group assortment considering the combination of the three T-scores, creating an ordinal variable of 4 levels as follows: T-score group: Group 3: all patients defined as osteoporotic (at least one T-score is ≤−2.5). Group 2: two T-score values are ≤0, and at least one is <−0.5. Group 1: two T-score values are positive or at least two T-score values with an absolute value <0.5. Group 0: all three T-score values are positive. The novel group division is also noted in the abovementioned file.
