# Supplementary material for: Quantitative MR Analysis of Changes in the Radius Bone Marrow in Osteoporosis
Source: J Osteoporos. 2023 Dec 27;2023:7861495. doi: 10.1155/2023/7861495 (PMC10764646; doi:10.1155/2023/7861495)
Supplement: Supplementary Materials — (1) A file including all clinical data of the participants is attached. (2) Scatterplots of the data depicted in Figures 2–4 are available in a separate file, to illustrate the variations within the groups. (3) Additional group assortment by T-scores: as mentioned, in the absence of a pre-existing weighted T-score in clinical use, we sought to create an additional group division in which the three T-scores are weighed. Therefore, we generated novel group assortment considering the combination of the three T-scores, creating an ordinal variable of 4 levels as follows: T-score group: Group 3: all patients defined as osteoporotic (at least one T-score is ≤−2.5). Group 2: two T-score values are ≤0, and at least one is <−0.5. Group 1: two T-score values are positive or at least two T-score values with an absolute value <0.5. Group 0: all three T-score values are positive. The novel group division is also noted in the abovementioned file. [file 7861495.f1.zip › Data_of_Examinees__.pdf]

Examinee	Study Group	P1NP	CTX	E2	LH	FSH	BMD HIP	T-score HIP	BMD FN	T-score FN	BMD LS	T-score SPINE	T-Score Group	T2	T1w	T1f	T1 FF	Fat % Dixon	Alcohol use	Family History	Hormonal Pathologies	Malignancy	Sedentary lifestyle	Calcium	Physical exercise	Smoking	BMI	Height	Weight	Age
1	2	69	582	30	25	65	0.91	-0.8	0.89	-0.8	1.45	2.1	2	119.08	1237	352	0.90		1	1	0	0	0	1	1	0	24.30	1.72	72	64
2	3	70	467	<20	49	95	0.85	-1.2	0.85	-1.1	0.88	-2.5	3	113.35	1115	350	0.88	92.67	0	1	0	0	1	1	0	0	25.70	1.58	61	59
3	1	49	350		6.4	4.8	0.99	0	0.94	-0.5	1.19	-0.1	1	120.77	1228	370	0.90	90.83	0	0	0	0	1	1	1	0	26.15	1.76	81	49
4	1	36	150	130	2.6	7.2	1.02	0.2	1.06	0.6	1.13	-0.6	1	115.72	1250	350	0.90	91.32								0	18.03	1.58	45	50
5	1			34	3	18	1.03	0.3	1.04	0.8	1.18	-0.1	1	117.60	1078	350	0.84	90.62							.	0	22.58	1.63	60	50
6	2						1.03	0.3	0.94	-0.3	1.32	1	1	118.57	1195	376	0.90	90.44	0	0	1	0	0	1	1	0	25.80	1.46	55	52
7	3	47	168	<20	33	67	0.66	-2.8	0.66	-2.7	0.83	-2.9	3	113.31	1221	354	0.88	94.27	0	0	0	0	1	1	1	0	25.22	1.63	67	67
8	3	57	477				0.67	-2.7	0.66	-2.7	1.00	-1.6	3	119.67	1172	362	0.95	92.96	0	1	0	0	0	0	0	0	21.91	1.57	54	62
9	2	48	450	22	34	125	1.2	1.7	1.03	0.4	1.31	0.9	0		1208	356	0.89	89.77	0	1	0	0	1	0	0	0	27.43	1.62	72	53
10	3	57	477	88	36	60	0.8	-1.7	0.84	-1.2	0.90	-2.5	3	119.24	1073	350	0.87	92.87	0	0	0	0	1	1	0	1	22.98	1.77	72	48
11	2	66	554	<20	24	67	1.01	0.1	0.99	0.1	1.37	1.4	0	115.31	1250	352	0.84	88.66	0	0	0	0	0	1	0	0	28.13	1.6	72	61
12	2			142	38	68	1.14	1.1	1.03	0.4	1.25	0.4	0	117.11	1235	350	0.89		1	1	0	0	0	1	0	0	18.29	1.62	48	47
13	2				27	67	0.85	-1.2	0.85	-1.1	1.10	-0.5	2	110.76	1233	354	0.87		0	Mother - Osteopenia	0	0	0	0	0	1	24.39	1.46	52	52
14	3	59	540	33	60	160	0.94	-0.5	0.84	-1.2	0.88	-2.7	3	120.52	1050	378	0.93	90.81							.	1	27.39	1.48	60	61
15	1	28	252	50	25	33	1.17	1.4	0.98	0	1.08	-0.9	2	122.63	1235	364	0.89	89.20	0	0	0	0	0	1	1	0	25.78	1.6	66	54
16	3	40	420	31	30	51	0.81	-1.6	0.77	-1.8	0.75	-3.6	3	116.88	1250	376	0.90	91.97	0	0	0	0	0	0	0	0	29.67	1.59	75	65
17	3	51	410	<20	11	60	0.67	-2.8	0.64	-2.9	0.81	-3.1	3	117.20	1220	382	0.91	90.14	0	0	0	0	0	1	1	0	23.27	1.565	57	66
19	2	52	357	32	20	64	0.98	-0.1	0.88	-0.8	1.11	-0.7	2	117.94	1250	374	0.89	93.99							.					62
20	2	38	421	137	30	36	1.19	1.5	1.10	1	1.25	0.4	0	119.16	1051	370	0.92	90.59	0	0	0	0	0	0	0	0	25.91	1.69	74	54
21	3	58	430	<20	21	74	0.8	-1.7	0.82	-1.3	0.87	-2.6	3					90.24	0	0	0	0	0	0	0	0	24.14	1.55	58	63
22	2			<20	18	40	0.97	-0.3	0.94	-0.3	1.25	0.4	1	119.24	1181	356	0.85	89.43	0	1	0	0	0	1	0	1	27.12	1.685	77	52
23	2						0.97	-0.2	0.99	0.1	0.99	-1.5	2	113.23	1050	362	0.91	89.72	1	0	0	0	1	0	0	1	21.45	1.7	62	48
26	1	34	201	63	8.6	14	1.1	0.8	1.03	0.4	1.46	2.1	0	119.36	1250	368	0.87	89.39	0	0	0	0	1	1	0	0	25.50	1.76	79	43
28	3						0.64	-3	0.65	-2.8	0.99	-1.6	3	116.37	1217	354	0.89	91.95							.	1	21.41	1.66	59	61
29	1						1.02	0.2	0.94	-0.3	1.16	-0.3	1	120.60	1173	350	0.90	90.59	0	0	0	0	0	0	1	0	20.83	1.58	52	46
30	1	30	216	<20	6	16	1.04	0.3	0.96	0	1.34	1.2	0	118.75	1248	366	0.87	91.10	0	0	0	0	1	1	0	0	26.67	1.62	70	48
