# Supplementary material for: Quantitative MR Analysis of Changes in the Radius Bone Marrow in Osteoporosis
Source: J Osteoporos. 2023 Dec 27;2023:7861495. doi: 10.1155/2023/7861495 (PMC10764646; doi:10.1155/2023/7861495)
Supplement: Supplementary Materials — (1) A file including all clinical data of the participants is attached. (2) Scatterplots of the data depicted in Figures 2–4 are available in a separate file, to illustrate the variations within the groups. (3) Additional group assortment by T-scores: as mentioned, in the absence of a pre-existing weighted T-score in clinical use, we sought to create an additional group division in which the three T-scores are weighed. Therefore, we generated novel group assortment considering the combination of the three T-scores, creating an ordinal variable of 4 levels as follows: T-score group: Group 3: all patients defined as osteoporotic (at least one T-score is ≤−2.5). Group 2: two T-score values are ≤0, and at least one is <−0.5. Group 1: two T-score values are positive or at least two T-score values with an absolute value <0.5. Group 0: all three T-score values are positive. The novel group division is also noted in the abovementioned file. [file 7861495.f1.zip › Scatter Plots.pdf]

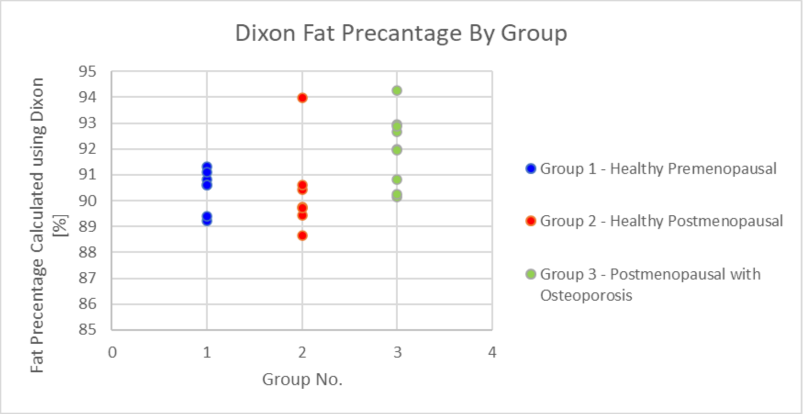

Figure 2.1

Scatter plot of the distribution of fat percentage values of the Radius BM according to the trial groups, as acquired by Dixon protocol. Fat percentage varied as follows: Healthy-premenopausal: 90.43±0.82 [%], Healthy-postmenopausal: 90.37±1.72 [%], Postmenopausal with Osteoporosis: 91.99±1.38 [%].

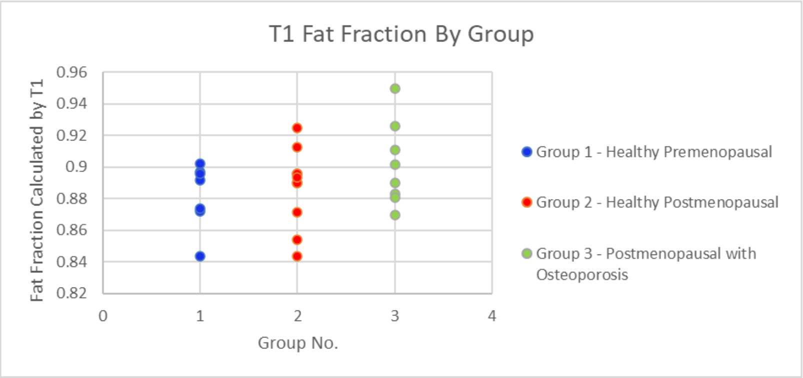

Figure 3.1

Scatter plot of the distribution of fat fraction values of the Radius BM, obtained by a two-compartment model of T1 relaxation time. Fat fraction varied as follows: Healthy-premenopausal: 0.88±0.02, Healthy-postmenopausal: 0.89±0.02, Postmenopausal with Osteoporosis: 0.90±0.03.

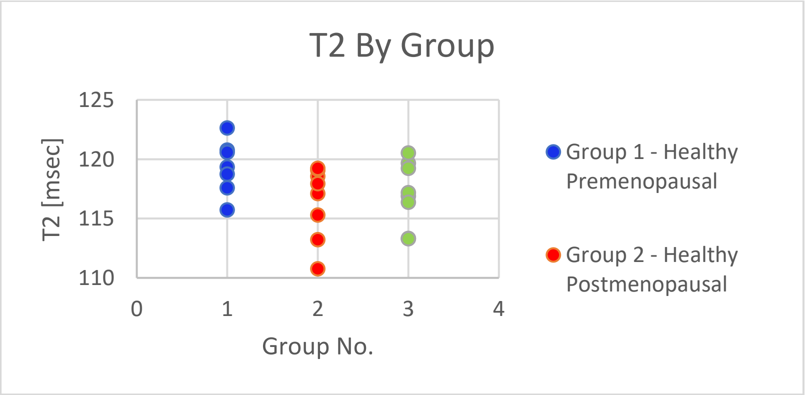

Figure 4.1

Scatter plot of the distribution of T2 values of the Radius BM by trial groups. T2 values varied as follows: Healthy-premenopausal: 119.35±2.27 [msec], Healthy-postmenopausal: 116.71±3.01 [msec], Postmenopausal with osteoporosis: 117.07±2.72 [msec].
